# Enzyme-instructed morphological transition of the supramolecular assemblies of branched peptides

**DOI:** 10.3762/bjoc.16.221

**Published:** 2020-11-04

**Authors:** Dongsik Yang, Hongjian He, Bing Xu

**Affiliations:** 1 Department of Chemistry, Brandeis University, 415 South Street, Waltham, MA 02454, USA

**Keywords:** acetylation, branched peptides, enzyme, nanostructures, N-terminal, responsive, self-assembly

## Abstract

Here, we report the use of an enzymatic reaction to cleave the branch off branched peptides for inducing the morphological transition of the assemblies of the peptides. The attachment of DEDDDLLI sequences to the ε-amine of the lysine residue of a tetrapeptide produces branched peptides that form micelles. Upon the proteolytic cleavage of the branch, catalyzed by proteinase K, the micelles turn into nanofibers. We also found that the acetylation of the N-terminal of the branch increased the stability of the branched peptides. Moreover, these branched peptides facilitate the delivery of the proteins into cells. This work contributes insights for the development of peptide supramolecular assemblies via enzymatic noncovalent synthesis in cellular environment.

## Introduction

Peptides, being able to self-assemble to exhibit emergent properties and functions [1–5], have received considerable attentions recently. For example, peptide assemblies are being explored for a wide range of applications, including cell cultures [6], tissue engineering [7], drug delivery [8–11], antibacterial agents [12–13], regarding biomineralization [14–15], as collagen mimics [16], anisotropic hydrogels [17–18], for cancer therapy [19–26], as mimicry of amyloids [27], in the context of intracellular phase transition [28], and in molecular imaging [29–30]. Most of these studies are centered on peptide amphiphiles or amphiphilic peptides that are linear in geometry. Nature, however, also produces and utilizes nonlinear peptides. For example, nonribosomal peptides exist in other geometries, such as branched (e.g., bleomycin) or cyclic peptides (e.g., vancomycin) [31]. While the understanding of the synthesis of branched peptides is well-developed, the self-assembly and enzymatic conversion of branched peptides has received limited attention [2,32–37]. For example, Stupp et al. reported that a cell adhesion epitope, RGDS, acts as a branch to peptide amphiphiles for making hydrogels via self-assembly [34,36]. Ulijn et al. connected Fmoc-DAARRGG to a lysine side chain for incorporation in a polymeric hydrogel for a responsiveness to thermolysin [35]. Dong et al. reported the branching of an oligopeptide via a cysteine linkage to result in multivalence [37]. During our studies on enzyme-instructed self-assembly (EISA) [38–40] of branched peptides for hydrogelation [32], we serendipitously found that an enzyme-responsive branched peptide was able to deliver small molecules or proteins to mitochondria efficiently in a cell-specific manner [33]. The branched peptide, which bears FLAG-tag as the branch [32], forms micelles. Certain proteases on the mitochondria of certain cells cleave the branch of the peptide, and such enzymatic proteolysis turns the micelles into nanofibers. This morphological transition (i.e., micelle to nanofiber) releases the cargos into the mitochondria of the cells [33]. These results imply that enzyme-responsive branched peptides can act as responsive biomaterials, especially in cellular environment. Thus, we decided to further explore the molecular space and cell compatibility of enzyme-responsive branched peptides.

**Figure 1:**
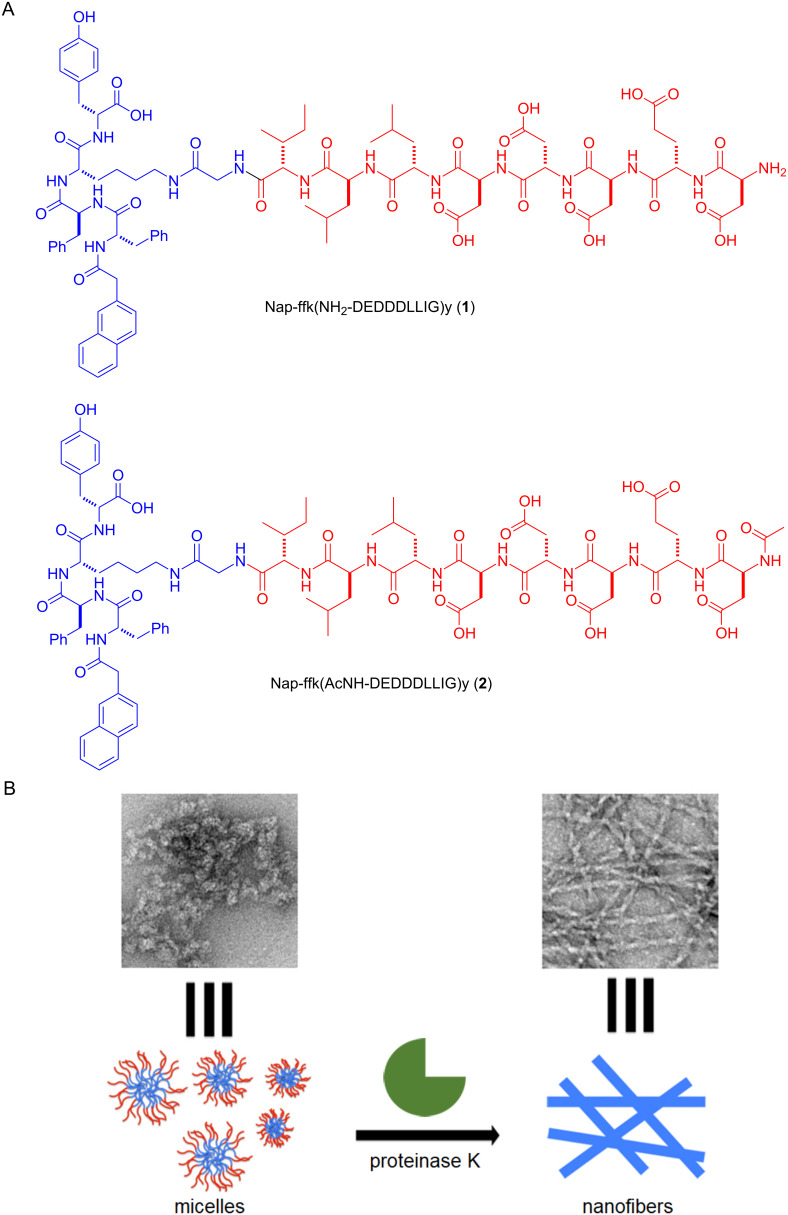
A) The molecular structures of the branched peptides Nap-ffk(NH_2_-DEDDDLLIG)y (**1**) and Nap-ffk(AcNH-DEDDDLLIG)y (**2**) for the enzymatic morphological transition. B) TEM images and the schematic illustration of the proteinase K-catalyzed transformation of the micelles of the branched peptide **1** to nanofibers.

Here, we report the use of an enzymatic reaction to cleave the branch off branched peptides for inducing the morphological transition of the assemblies of the peptides. The conjugation of Asp–Glu–Asp–Asp–Asp–Leu–Leu–Ile–Gly (DEDDDLLIG) sequences to the ε-amine of the lysine residue of a tetrapeptide Nap–ᴅ-Phe–ᴅ-Phe–ᴅ-Lys–ᴅ-Tyr (Nap-ffky) [9] produces the branched peptide **1**, which forms micelles (Figure 1). When proteinase K catalyzed the proteolysis to cleave the branch off **1**, the micelles turn into nanofibers. Acetylation of the N-terminal of the branch forms **2**, which exhibits an enhanced stability towards the proteolysis. In addition, the cleavage also occurs at other sites in the branch of **2**, albeit less frequently, due to the acetylation. Both **1** and **2**, being synthesized for the first time, are able to facilitate the delivery of the proteins into cells. This work, illustrating enzymatic debranching to control the morphology of peptide assemblies, contributes to the development of peptide supramolecular assemblies via enzymatic noncovalent synthesis [41–42] in a cellular environment.

## Results and Discussion

### Molecular design

We designed the branched peptides including the DEXXXLLI sequences [43] for this study. The DEXXXLLI (X is any amino acid) sequences are sorting signals of adaptor protein (AP) complexes, which play a critical role in endocytic pathways. Figure 1A shows the molecular design of the branched peptides, which also act as hydrogelator precursors [44–45]. The branch consists of (i) a hydrophilic ʟ-peptide segment for enzymatic recognition and cleavage, (ii) a self-assembling ᴅ-peptide sequence composed of a 2-acetylnaphthyl group and a tetrapeptide (ᴅ-Phe–ᴅ-Phe–ᴅ-Lys–ᴅ-Tyr), and (iii) a glycine as the spacer for the incorporation of (i) and (ii). For the hydrophilic ʟ-peptide, DDD substitutes the XXX site in the DEXXXLLI sequence to increase the hydrophilicity of the peptides. In order to enable the self-assembly of branched peptides after the enzymatic reaction, we used Nap–ᴅ-Phe–ᴅ-Phe (Nap-ff) to provide aromatic–aromatic interactions from diphenylalanine for self-assembly because Nap-ff is a versatile motif to enable the self-assembly of many small molecules [46]. More specifically, we choose Nap-ffky since lysine allows the conjugation of the cleavable side chain, and tyrosine provides additional aromatic–aromatic interactions, as shown in a recent cryo-EM structure (PDB: 6X5I) [41]. Based on the above rationale, the DEDDDLLIG sequences attach to the ε-amine of the lysine residue of the tetrapeptide Nap-ffky to form the branched peptide **1**. To investigate how the acetylation of aspartic acid affects the proteolysis and the formation of assemblies, the acetylation of the N-terminal of the branch in **1** would generate **2**.

### Synthesis

**Scheme 1:**
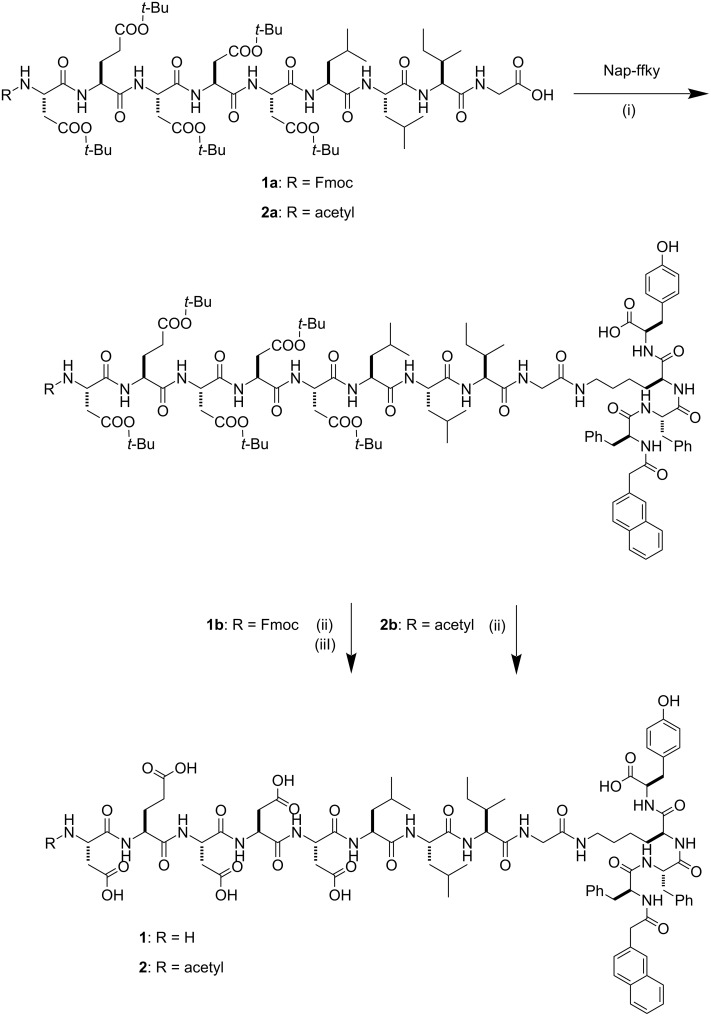
Synthetic route to the branched peptides. (i) HBTU, DIPEA, DMF, 12 h, rt, (ii) TFA, 2 h, rt, and (iii) 20% piperidine in DMF, 2 h, rt.

We used 2-chlorotrityl chloride resin for the typical Fmoc solid-phase peptide synthesis (SPPS) [47] to produce the peptides shown in Scheme 1. We first synthesized the peptide segments (i.e., Fmoc-DEDDDLLIG (**1a**) and acetyl-DEDDDLLIG (**2a**)). We kept the *tert*-butyl protecting groups of aspartic acid for the coupling reaction with Nap-ffky. We used 2,2,2-trifluoroethanol (TFE) in dichloromethane for 2 h to cleave the crude peptides from the resin. After using diethyl ether to precipitate the crude protected peptides, we used HBTU in the presence of DIPEA in DMF to react the peptides with Nap-ffky overnight. Then, we removed the Fmoc-protection for the branched peptide **1b**. As a final step, we used TFA at room temperature for 2 h to cleave the *tert*-butyl protecting groups of **1b** and **2b**. After adding diethyl ether to precipitate the crude peptides, centrifugation, and washing three times, we used reversed-phase HPLC and acetonitrile (containing 0.1% TFA) and double-distilled water (containing 0.1% TFA) as the eluents to purify the designed branched peptides **1** and **2**.

### Enzymatic reaction of the branched peptides

**Figure 2:**
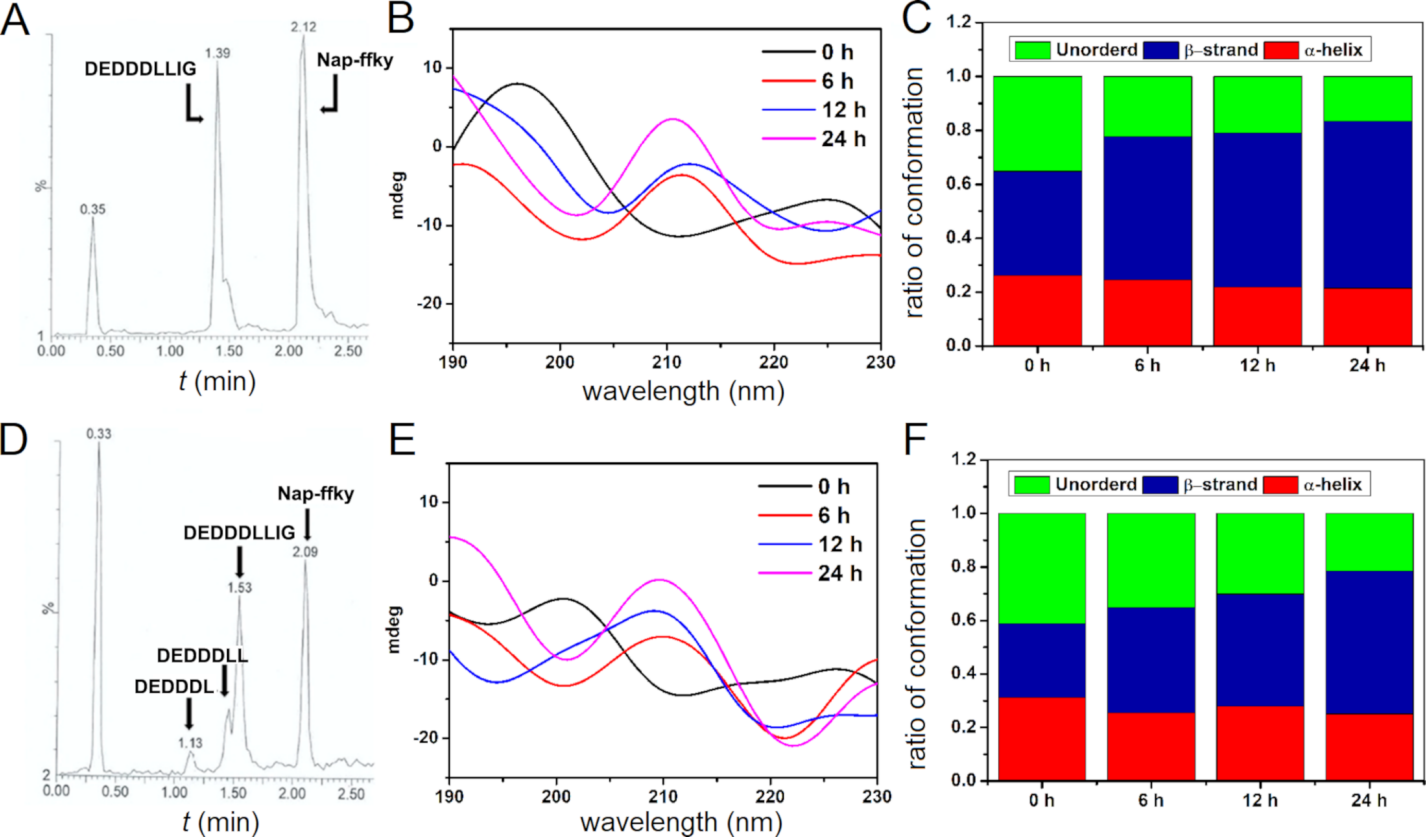
A) LC–MS spectrum of the proteolytic products after **1** (5 mM) was incubated with proteinase K (5 U/mL) for 24 h. B) Time-dependent circular dichroism (CD) spectra of **1** incubated with proteinase K in Tris-HCl buffer at pH 7.0. C) CD simulation of the mixture from the spectra in B. D) LC–MS spectrum of the proteolytic products after **2** (5 mM) was incubated with proteinase K (5 U/mL) for 24 h. E) Time-dependent CD spectra of **2** incubated with proteinase K in Tris-HCl buffer at pH 7.0. F) CD simulation of the mixture from the spectra in E.

After having obtained the peptides **1** and **2**, we examined the enzymatic proteolysis of **1** and **2** catalyzed by proteinase K. We added proteinase K, a powerful endopeptidase, to the solutions of **1** and **2** (5 mM, pH 7.0, in Tris-HCl buffer). Our results show that the branched peptide **1** undergoes proteolytic cleavage to form DEDDDLLIG and Nap-ffky after 24 h, while **2** proteolytically hydrolyzes into four parts, including DEDDDLLIG, DEDDDLL, DEDDDL, and Nap-ffky. These results indicate that the cleavage occurs depending on the acetylation of the N-terminal of the branch. Then, we conducted a time-dependent CD analysis to examine the change of the secondary structural conformations of **1** and **2** during the proteolysis catalyzed by proteinase K in Tris-HCl buffer at pH 7.0. As shown in Figure 2B and Figure 2E, the CD spectra of **1** and **2** exhibit a negative peak at 210 nm, suggesting that the peptides can adopt a β-strand conformation to form nanofibrils. During the transition from micelles to fibrils, the CD signal at 210 nm reverses the sign, likely due to the self-assembly of Nap-ffky to helical fibrils. Analyzing the CD spectra by the Contin-LL analysis program [48], we estimated the percentage of each conformation, further confirming the trend and the assignment of the dominant conformation. As shown in Figure 2C and Figure 2F, the initial state of peptide **1** has more β-strand conformations than that of peptide **2**. With increasing proteolysis time, the CD spectra indicate that the cleavage of **1** or **2** results in more assemblies that favor β-strand conformations (Figure 2D and Figure 2E). In addition, the ratio of the β-strand conformation resulted from the cleavage of **1** being slightly faster than that of **2**.

### Enzymatic hydrogelation and morphological transition

**Figure 3:**
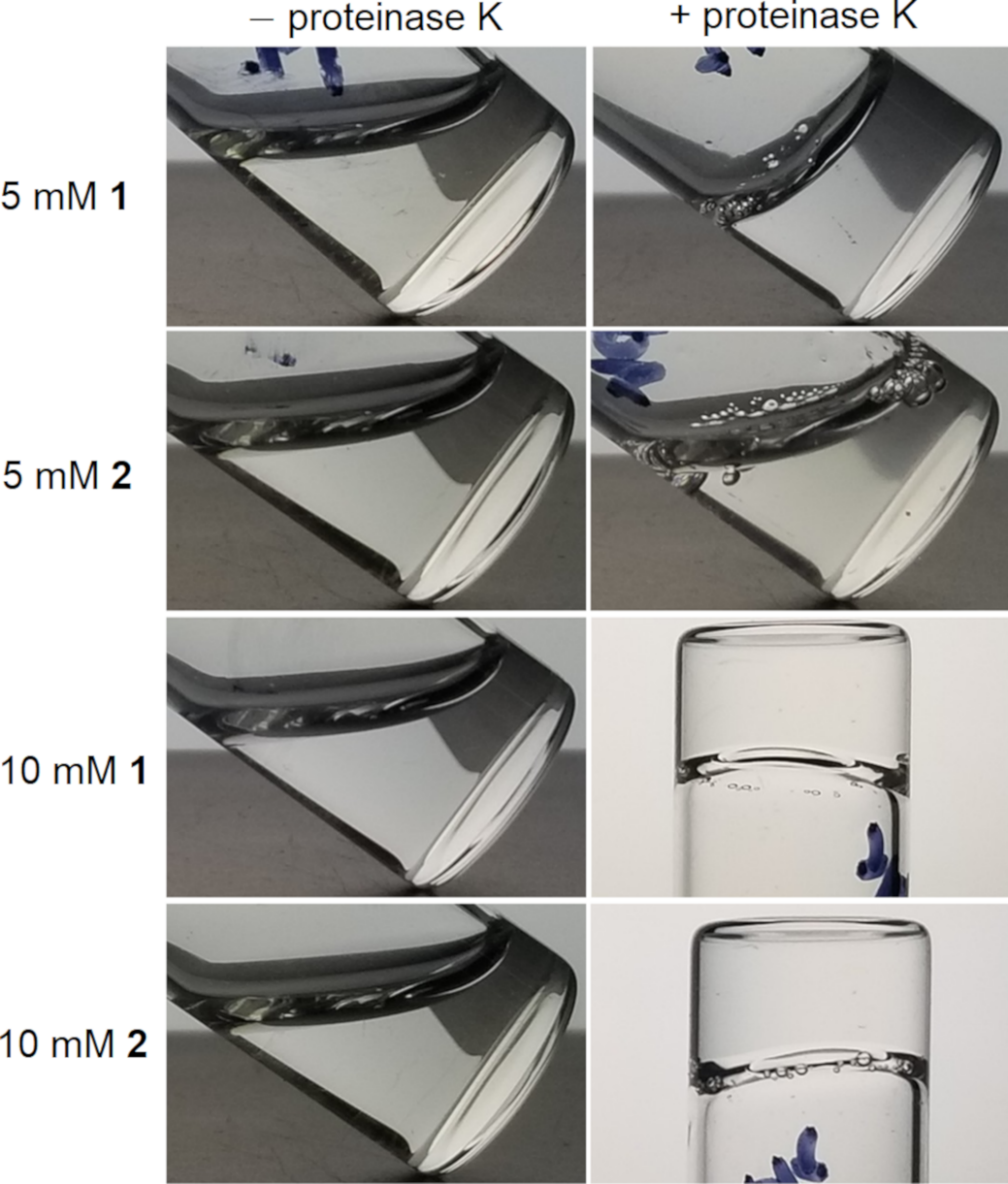
Optical images of solutions of **1** and **2** (5 mM), respectively, with or without proteinase K (5 U/mL) or of **1** and **2** (10 mM), respectively, with or without proteinase K (10 U/mL) after 24 h in Tris-HCl buffer at pH 7.0.

We evaluated the enzymatic hydrogelation of **1** and **2** upon the proteolysis catalyzed by proteinase K. As shown in Figure 3, **1** or **2** form a clear solution in Tris-HCl buffer. The addition of proteinase K (5 U/mL) to a solution of **1** (5 mM) results in a weak gel, while the addition of proteinase K (5 U/mL) to a solution of **2** (5 mM) only produces a viscous solution. The addition of an increased amount of proteinase K (10 U/mL) to a solution of **1** (10 mM) or **2** (10 mM) results in a stable hydrogel. These results indicate that hydrogelation occurs after proteinase K catalytically produces more Nap-ffky for self-assembly. In addition, unlike the FLAG-tag-based branched peptides [32], **1** and **2** are proteolytic-resistant to enterokinase (ENTK, Figures S3 and S4, Supporting Information File 1).

**Figure 4:**
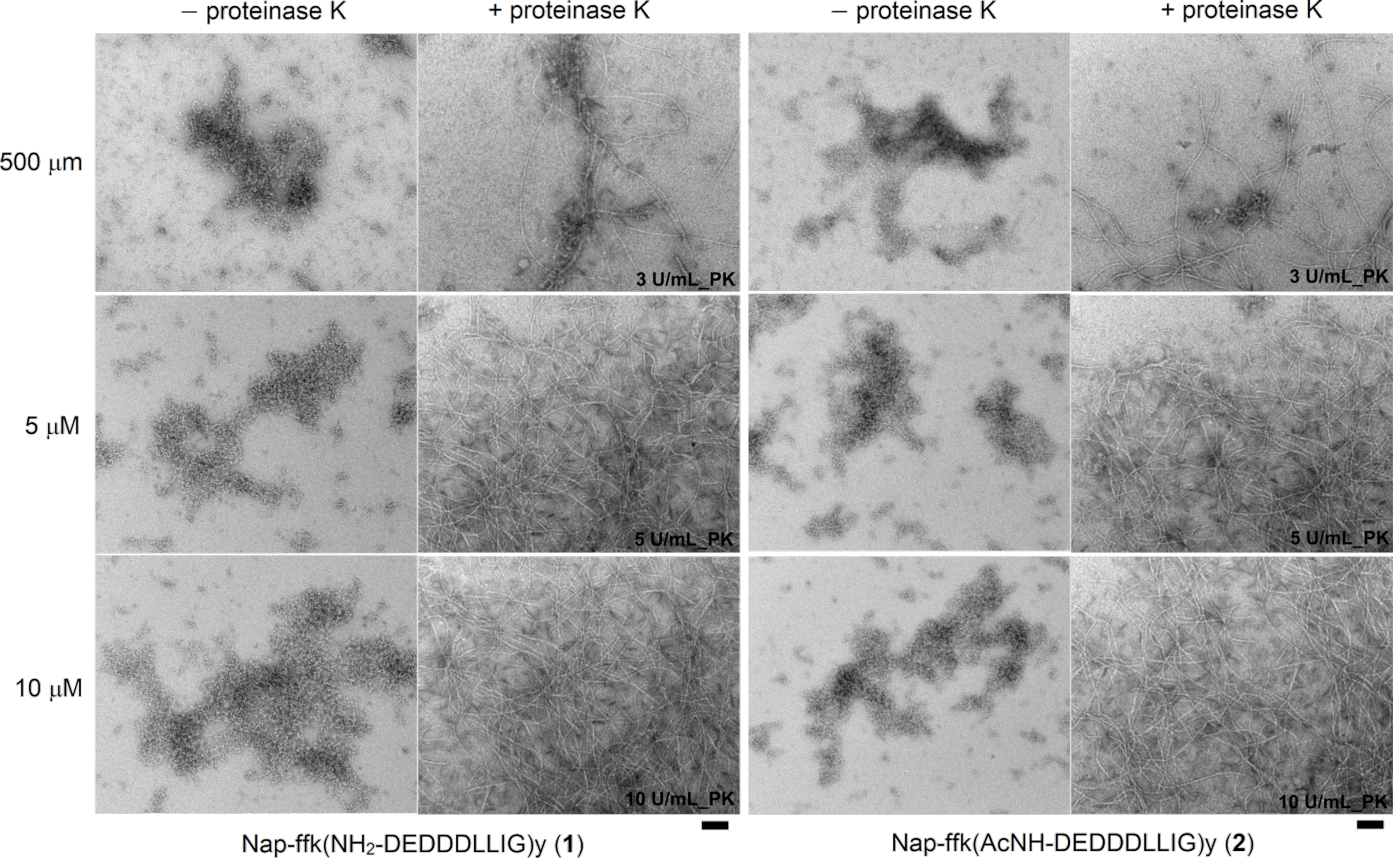
TEM images of the branched peptides before and after the addition of proteinase K into the solutions of **1** or **2** in tris-HCl buffer, after 24 h at pH 7.0. Scale bar = 100 nm. Left: peptide **1**, right: peptide **2**.

We used TEM to examine the nanoscale morphology changes before and after the addition of proteinase K to solutions of **1** and **2**. TEM revealed the solutions of **1** and **2** (500 μM) to contain nanoparticles of 11 ± 2 nm in diameter, indicating that the acetylation at the branch affects the self-assembly of the branched peptides only slightly. Upon the addition of proteinase K, the nanoparticles transformed to nanofibers of 12 ± 2 nm in diameter (Figure 4). This result indicates that the branched peptides form micelles in Tris-HCl buffer, and proteinase K generates Nap-ffky from **1** or **2**, resulting in the nanofibers. When the concentration of **1** or **2** was increased to 5 mM and 10 mM, TEM revealed that the solutions contained nanoparticles, suggesting that the negatively charged branches in the micelles prevent the formation of worm-like micelles due to increasing the concentration of **1** or **2**. After the addition of proteinase K to the solution of **1** (5 mM) or **2** (5 mM), the hydrogel of **1** contains long, flexible nanofibers with a diameter of 12 ± 2 nm. Similarly, the viscous solution of **2** also consists of nanofibers with a width around 12 nm. When the concentration of **1** or **2** is at 10 mM, the addition of proteinase K also generates the nanofibers at a diameter of 12 ± 2 nm, but with a considerable number of bundles and increasing degree of entanglement, which reflects the significant interfiber interactions due to the increase of the concentration of **1** or **2**. Notably, the nanofibers, formed from cleaving the branch of the peptides at different concentrations, exhibit the same morphological appearances, agreeing with the statement that the nanofibers likely are made of Nap-ffky.

### Cytotoxicity, cell lysates, and protein delivery

**Figure 5:**
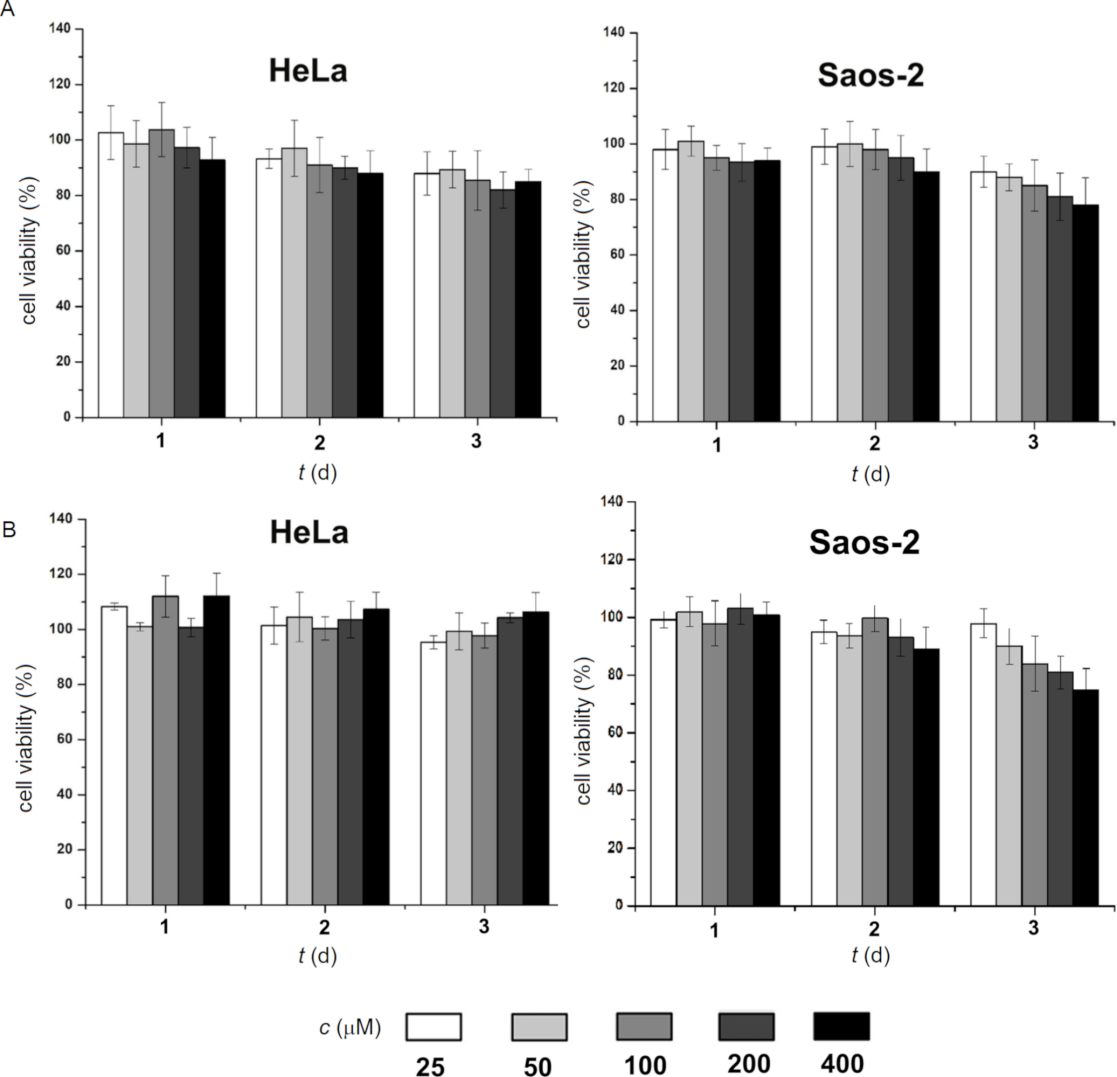
Cell viability of HeLa and Saos-2 cells treated with **1** (A) and **2** (B).

**Figure 6:**
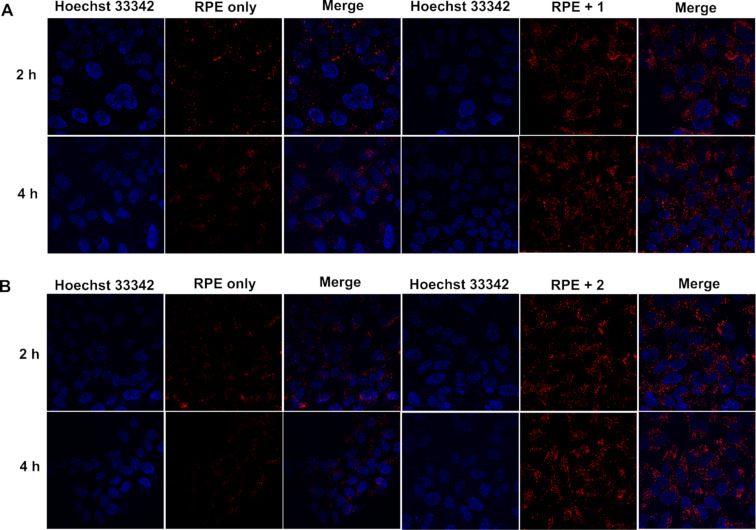
Confocal images of HeLa cells treated with a mixture of RPE (8 μg/mL) and A) **1** (400 μM) or B) **2** (400 μM) after 2 h and 4 h of incubation. Blue: nucleus, red: RPE.

We investigated the cell compatibility of **1** and **2** by incubation with two kinds of mammalian cells, HeLa and Saos-2 cells, using the 3-(4,5-dimethylthiazol-2-yl)-2,5-diphenyl tetrazolium bromide (MTT) assay. The cell viability of HeLa and Soas-2 cells after being incubated with **1** (400 μM) for three days are about 90% and 80%, respectively. In addition, HeLa and Soas-2 cells also exhibited 100% and 75% cell viability, respectively, after being incubated with **2** for 3 days (Figure 5). These results confirm that the branched peptides largely are innocuous to mammalian cells. To confirm that **1** and **2** convert from micelles to nanofibers in a cellular environment, we incubated of **1** or **2** at 500 μM in the lysates of HeLa cells (Figure S5, Supporting Information File 1) and examined the amount of each component in % by LC–MS. After 24 h incubation, 44% of **1** or 39% of **2**, was cleaved to Nap-ffky, which self-assembles as part for the nanofibers. These results indicate that the assemblies of the branched peptides transform to the nanofibers in the HeLa cells. Then, we used confocal laser scanning microscopy (CLSM) to examine whether these compounds deliver RPE into HeLa cells. After mixing R-phycoerythrin (RPE) [49], a red fluorescent protein, with **1** or **2** at different concentrations, we incubated the mixtures with HeLa cells for 2 h and 4 h. While the control cells (without adding **1** or **2**) showed little fluorescence, the cells treated with RPE and **1** or **2** started to show many red fluorescent puncta inside the cells after 2 h. The puncta remained inside the cells at 4 h and showed little change (Figure 6). These results indicate that the micelles formed by **1** or **2** are able to deliver protein cargos into HeLa cells and that the delivery apparently reaches a steady state after 2 h because further increasing the time barely enhances the delivery.

## Conclusion

In summary, this work demonstrates the branched peptides as novel molecular precursors for enzymatic self-assembly and phase transition. The morphological transformation of the negatively charged branched peptides in solution from micelles to nanofibers upon proteolysis suggests that it is feasible to explore other hydrophilic branches for branched peptides to undergo enzymatic self-assembly. Moreover, our results show that the acetylation of the N-terminal of the branch retards the proteolysis, which should provide an additional handle to tune the stability of the branched peptides in a cellular environment. This property may lead to branched peptides to respond to a specific protease, and thus providing a useful molecular platform for exploring the applications of enzyme-instructed self-assembly for soft materials.

## Supporting Information

File 1 Experimental part.
