# Supplementary material for: Enzyme-instructed morphological transition of the supramolecular assemblies of branched peptides
Source: Beilstein J Org Chem. 2020 Nov 4;16:2709–18. doi: 10.3762/bjoc.16.221 (PMC7653338; doi:10.3762/bjoc.16.221)
Supplement: File 1 — Experimental part. [file Beilstein_J_Org_Chem-16-2709-s001.pdf]

BEILSTEIN JOURNAL OF ORGANIC CHEMISTRY

Supporting Information

for

Enzyme-instructed morphological transition of the supramolecular assemblies of branched peptides

Dongsik Yang, Hongjian He and Bing Xu

Beilstein J. Org. Chem. **2020,** *16,* 2709–2718. doi:10.3762/bjoc.16.221

Experimental part

License and Terms: This is a supporting information file under the terms of the Creative Commons Attribution License (https://creativecommons.org/licenses/by/4.0). Please note that the reuse, redistribution and reproduction in particular requires that the authors and source are credited.
The license is subject to the *Beilstein Journal of Organic Chemistry* terms and conditions: (https://www.beilstein-journals.org/bjoc)

Experimental part

Materials and instruments: All chemical reagents and solvents were used directly as received from the commercial sources without further purification. The confocal laser-scanning microscopy (CLSM) images were obtained on a ZEISS LSM 880 confocal laser scanning microscope. Transmission electron microscopy imaging was conducted on a Morgagni 268 transmission electron microscope. LC–MS were taken with a Waters Acquity Ultra Performance LC with Waters a MICRO-MASS detector. Circular dichroism spectra were measured on a Jasco J-810 spectropolarimeter.

Solid-phase peptide synthesis: All the peptides shown in Scheme 1 were prepared by typical Fmoc solid-phase peptide synthesis (SPPS) using 2-chlorotrityl chloride resin and the corresponding Fmoc-protected amino acids with side chains properly protected. The first amino acid was loaded onto the resin at about 1.5 mmol/g of resin. After loading the first amino acid to the resin, the capping regent (DCM/MeOH/DIPEA 17:2:1, v/v/v) was used to ensure that all the active sites of the resin were protected. 20% piperidine in DMF was used to remove the Fmoc groups, the next Fmoc-protected amino acid was coupled to the free amino group using HBTU as the coupling reagent. The growth of the peptide chain followed the established Fmoc SPPS protocol. The crude peptides were collected using a TFA- or TFE-mediated cleavage method: The peptide derivatives were cleaved using the TFA cleavage solution (TFA/TIS/H_2O 95:2.5:2.5, v/v/v) or the TFE cleavage solution (20% TFE in DCM) for 1 h. After removing the cleavage solution, 20 mL of ice-cold ethyl ether per gram of resin was then added to the above solution. After discarding most of the supernatant, the resulting precipitate was filtered and dried by a lyophilizer.

All crude peptide compounds were purified by using reversed-phase HPLC (Agilent 1100 Series) equipped with an XTerra C18 RP column. We used acetonitrile (Fisher Scientific, HPLC grade) plus 0.1% trifluoroacetic acid (TFA) and water (Fisher Scientific, HPLC grade) plus 0.1% TFA as eluent.

Circular dichroism (CD) analysis: CD spectra were recorded (190−230 nm) using a JASCO 810 spectrometer under a nitrogen atmosphere. The peptide solution (500 μM) was placed evenly on the 1 mm-thick quartz cuvette and scanned with 0.5 nm intervals for three times. The percentage of the secondary structures in different samples was calculated by the programs provided in DichroWeb.

Cell culture: All cell lines were purchased from the American Type Culture Collection (ATCC). The HeLa cells were cultured in Minimum Essential Media (MEM) supplemented with 10% fetal bovine serum (FBS) and antibiotics. The HepG-2 cells were propagated in Dulbecco's Modified Eagle's Medium (DMEM), supplemented with 10% FBS and antibiotics. The Saos-2 cells were cultured in McCoy's 5A supplemented with 15% FBS and antibiotics. All cells were incubated in a fully humidified incubator containing 5% CO_2 at 37 °C.

Cell viability assay: The cells were seeded in 96-well plates with the density of 1×10^4 cells per well. The cells were allowed to attach to the wells for 24 h at 37 °C (5% CO_2). After the removal of the culture medium, we added the 100 μL culture medium containing compound **1** or **2** (5 concentrations) to each well. At a designated time (24/48/72 hours), 10 μL of an MTT solution were added to each well, and the plated cells were incubated for 4 h. Then, 100 μL of sodium dodecyl sulfate (SDS) were added to quench the reduction

reaction and to dissolve the purple formazan. After incubation of the cells at 37 °C overnight, the absorbance of each well at 595 nm was measured in a multimode microplate reader. Three independent experiments were performed, with an average ± standard deviation.

Confocal microscopy: Cells in the exponential growth phase were seeded in a glass-bottomed culture chamber at 1×10^5 cells/well. The cells were allowed to attach for 24 h at 37 °C (5% CO_2). After the removal of the culture medium, the cells were incubated with 8 μg/ml R-phycoerythrin (RPE) with 400 μM of **1** or **2** for 2 h and 4 h, respectively. A new culture medium containing the desired concentrations of the compound was added. After rinsing the cells three times with live cell imaging buffer, the cells were imaged immediately using a Zeiss 880.

Transmission electron microscopy: The 400 mesh copper grids coated with a continuous thick carbon film (≈35 nm) were glowed. We placed 5 μL of the sample solution on the grids and incubated for 45 s at room temperature. Then, we rinsed the grid with double-distilled water twice, and the grids containing the sample were stained with 2.0% w/v uranyl acetate two times. The grids were allowed to dry for imaging. TEM images were taken with a Morgagni 268 transmission electron microscope.

Preparation of cell lysate:

Cell lysis buffer (10X, nondenature) was purchased from Cell Signaling Technology.

1. Seed cells in a 10 cm petri dish and wait for confluency.

2. Add trypsin to collect the cells and wash the plate with PBS to remove residual medium.

3. Centrifuge at 1,000 x g for 3 minutes at 25 °C.

4. Add 1 mL of 1X lysis buffer into the tube.

5. Apply 5 freeze–thaw cycles.

6. Centrifuge at 12,000 x g for 10 minutes at 4 °C.

7. Collect the supernatant for use, and add 500 μM of **1** or **2**.

LC–ESIMS

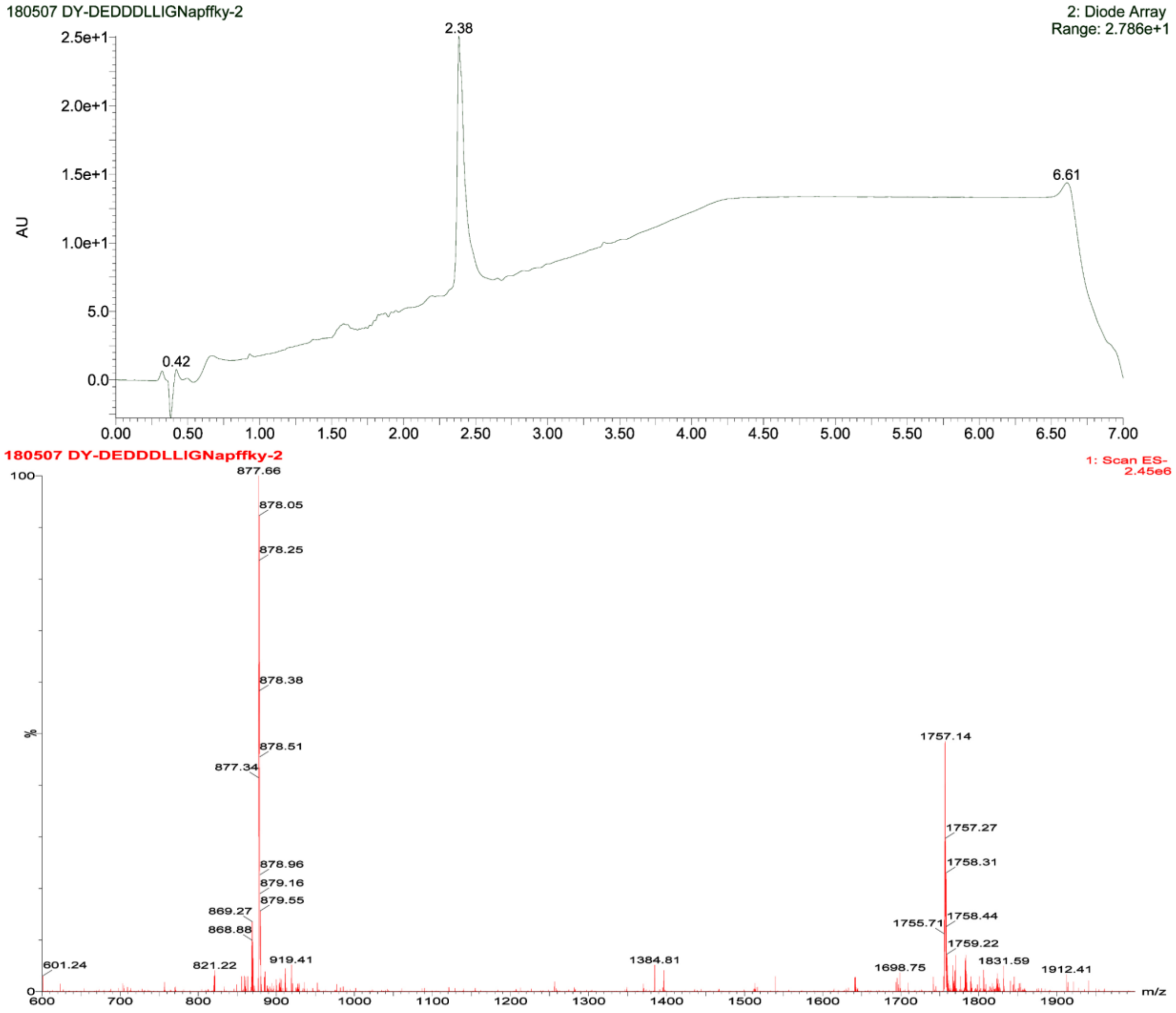

Figure S1. LC–MS spectrum of **1** (*m*/*z*): $C_{86}H_{112}N_{14}O_{26}$, calc. *M* = 1756.79; observed = 1757.14).

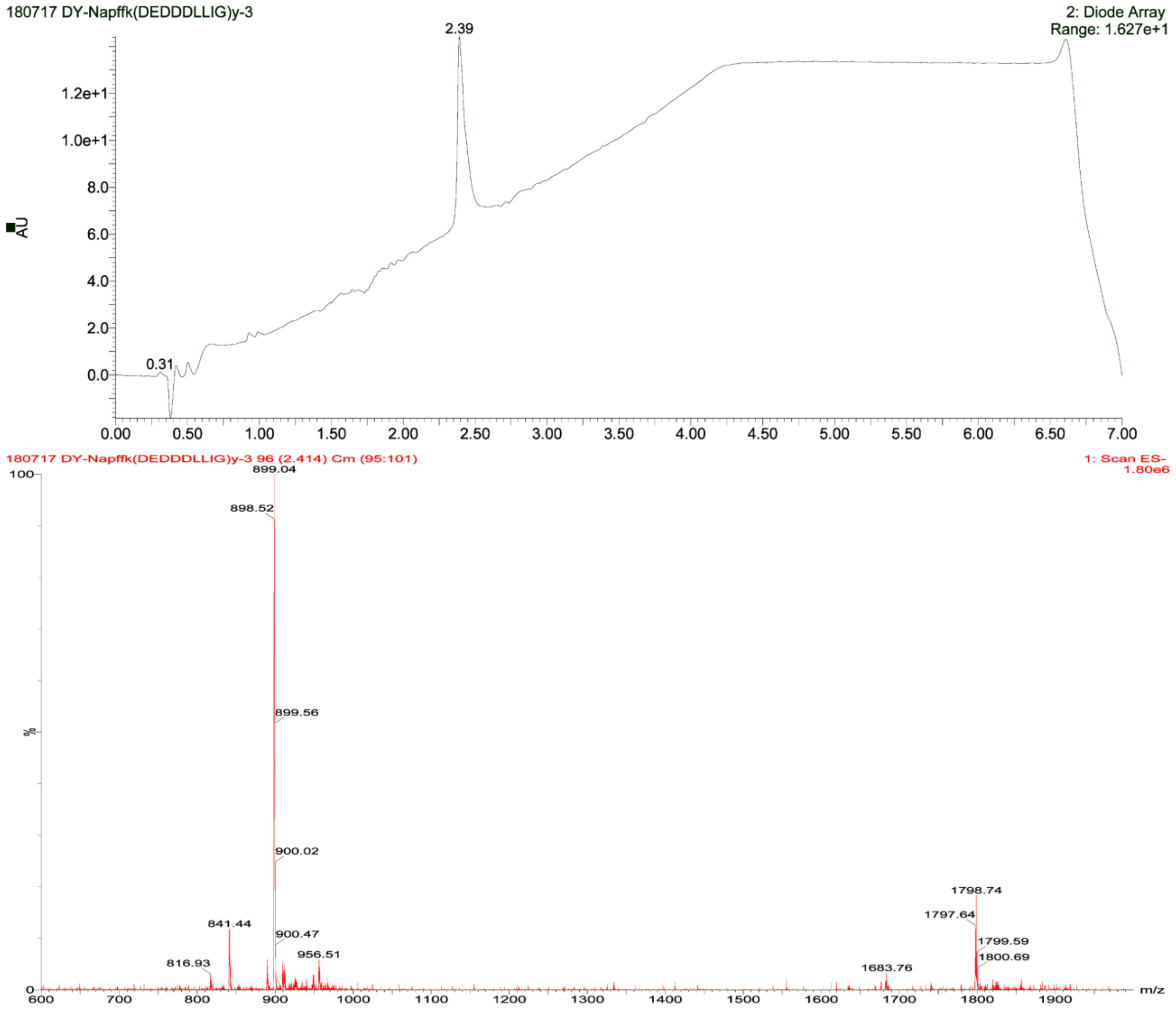

Figure S2. LC–MS spectrum of **2** (*m*/*z*): $C_{88}H_{114}N_{14}O_{27}$, calc. *M* = 1798.80; observed = 1798.74.

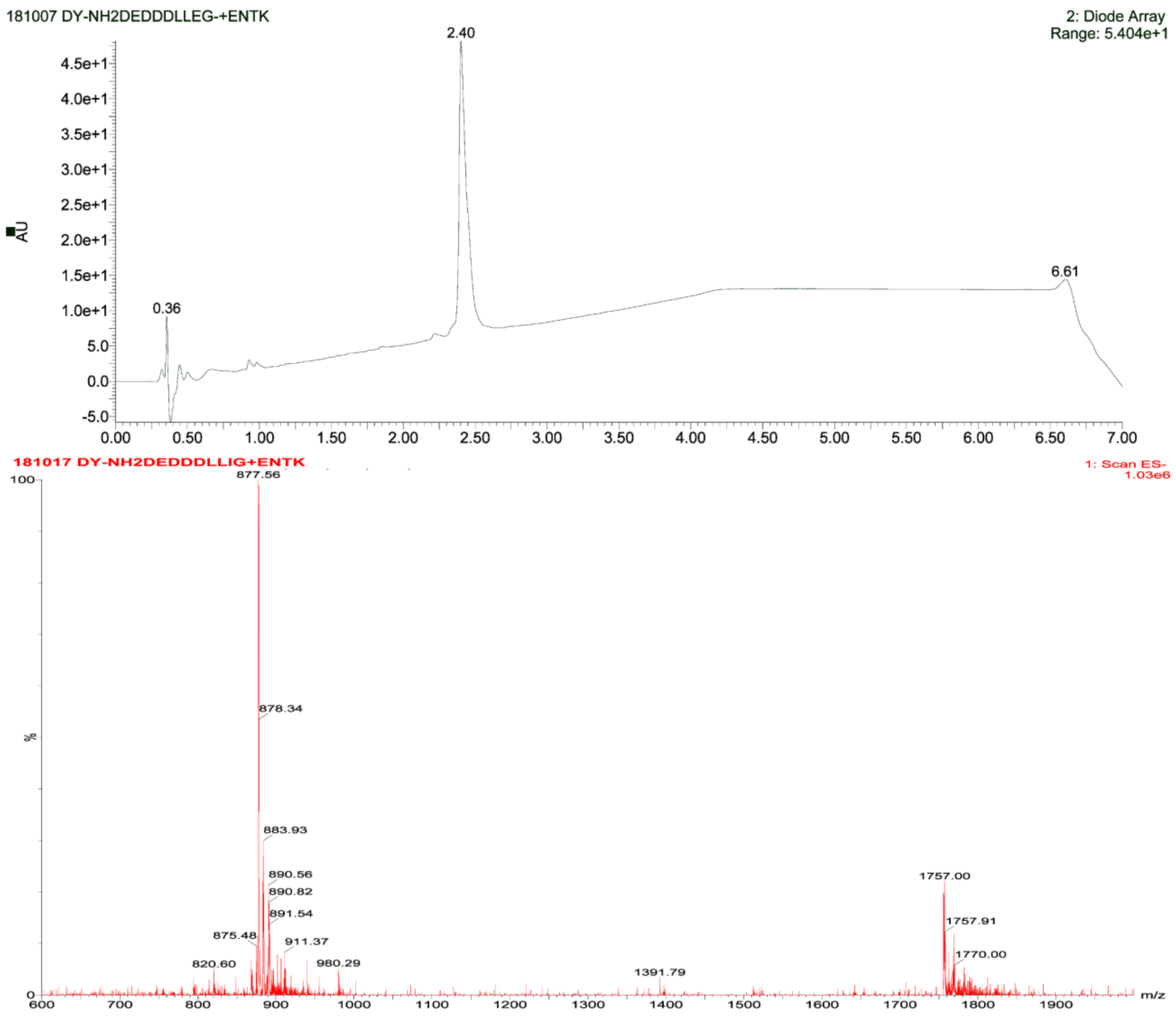

Figure S3. LC–MS of the **1** (M = 1756, 2.5 wt %) by addition of ENTK (10 U/ml) after 24 hours at 37 °C in PBS buffer at pH 7.4.

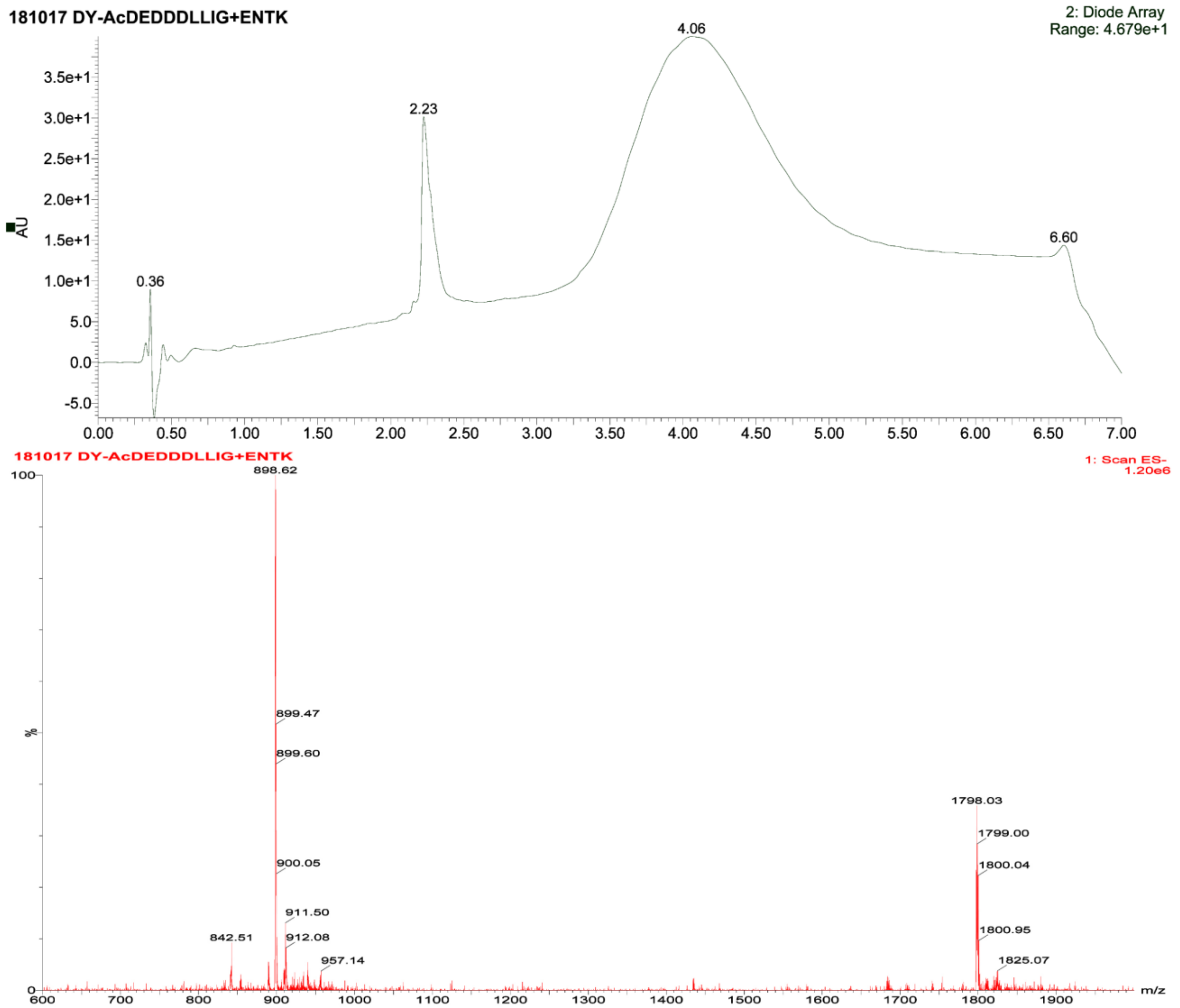

Figure S4. LC–MS of the **2** (*M* = 1798, 2.5 wt %) by addition of ENTK (10 U/ml) after 24 hours at 37 °C in PBS buffer at pH 7.4.

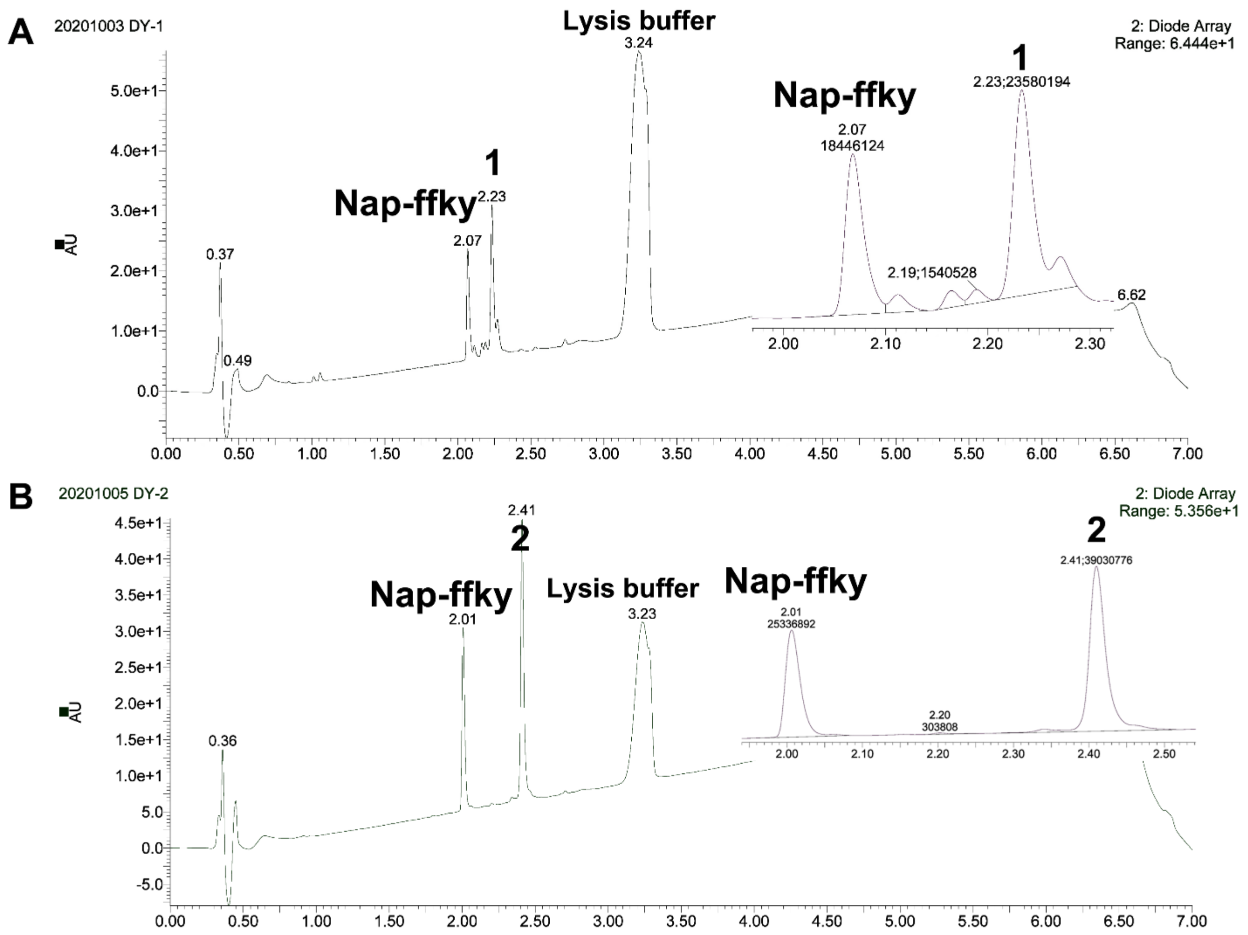

Figure S5. LC–MS evidences of the cell lysate of A) **1** and B) **2**. 500 μM of the peptides were incubated by cell lysate after 24 hours at 37 °C in 10 mmol of lysis buffer at pH 7.4.

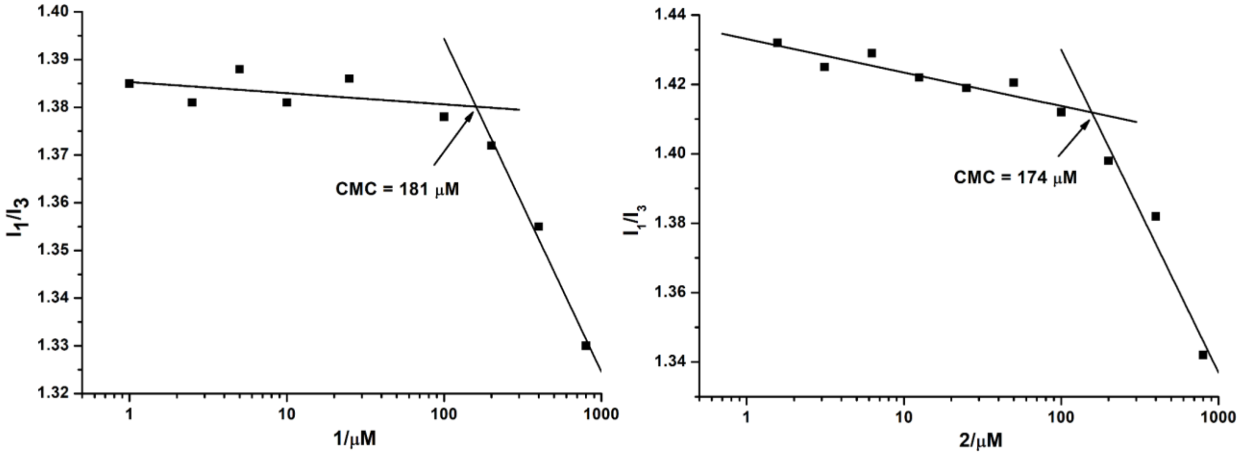

Figure S6. CMCs of **1** and **2** in PBS buffer (pH 7.4)
